# Bardet-Biedl syndrome in a 19-year-old male: the first case report from Palestine

**DOI:** 10.3389/fped.2024.1420684

**Published:** 2024-06-11

**Authors:** Hamza B. Karmi, Yahya Abu Jwaid, Mohammad Hakam Shehadeh, Dareen Njoom, Aya Awwad, Hasan Eideh

**Affiliations:** ^1^ Medical Research Club, Faculty of Medicine, Al-Quds University, Jerusalem, Palestine; ^2^ Department of Pediatric Endocrinology, Palestine Medical Complex (PMC), Ramallah, Palestine

**Keywords:** Bardet-Biedl syndrome, type 2 diabetes, retinitis pigmentosa, *FBN3* gene, Palestine

## Abstract

Bardet-Biedl syndrome (BBS) is a rare genetic disorder characterized by retinitis pigmentosa, polydactyly, type 2 diabetes mellitus, and obesity. This case report presents a 19-year-old male from Palestine with BBS, exhibiting delayed diagnosis and variable phenotypic expression. The patient had familial BBS history and presented with obesity, type 2 diabetes mellitus, retinitis pigmentosa, and cryptorchidism. Genetic analysis identified heterozygous missense variants in the *FBN3* gene, yet additional genetic factors may contribute to the phenotype. Renal abnormalities included kidney shrinkage and mild hydronephrosis. Management of this patient involves a multidisciplinary approach with lifestyle modifications, surgical interventions, and supportive care. Early diagnosis, genetic counseling, and regular follow-up are crucial for improving outcomes in BBS. This report highlights diagnostic and therapeutic challenges and underscores the need for further research on this complex disorder.

## Introduction

Bardet-Biedl syndrome (BBS) was independently described by both George Bardet and Arthur Biedl in 1920 and 1922, respectively (1, 2) BBS is a genetically heterogeneous disorder characterized by a wide spectrum of clinical manifestations and complications, integral to understanding its impact on affected individuals. The hallmark features of BBS include rod-cone dystrophy leading to progressive vision loss, obesity, polydactyly, renal abnormalities, learning difficulties, and hypogenitalism (3). Renal dysfunction, in particular, is a critical aspect of BBS, as it is the primary cause of morbidity and mortality in affected individuals (4). The syndrome also encompasses additional features such as metabolic disorders including insulin resistance and type 2 diabetes mellitus, as well as cardiovascular anomalies. The complexity of BBS is further underscored by its genetic basis, with mutations in multiple genes related to the structure and function of primary cilia contributing to its pathogenesis (4). The inheritance pattern is predominantly autosomal recessive, though more complex oligogenic inheritance has been observed, indicating the involvement of mutations in multiple genes (5).

The condition exhibits a notably high prevalence in Newfoundland and Kuwait, which is attributed to increased consanguinity within these populations (6), however, this disease is rare in other parts of the world and affects fewer than 5,000 people in the U.S (3, 7). Although diagnosis of this syndrome can be highly suggestive based on the characteristic clinical features, genetic analysis is necessary for confirmation. There is no specific treatment available for BBS; however, it is crucial to focus on managing its symptoms and preventing complications, as the disease only becomes life-threatening in advanced stages. The prognosis of BBS varies depending on the severity and progression of symptoms, especially kidney and heart involvement (8).

In this report, we present a case of a 19-year-old male patient who was diagnosed with BBS based on his clinical presentation and genetic analysis. After carefully reviewing the literature, this case will be the first case of this syndrome to be published from Palestine. We also briefly provide an overview of the current state of knowledge regarding this syndrome. By publishing this case, we aim to increase the awareness and knowledge of this rare disorder among clinicians and researchers and to highlight the challenges and opportunities for improving the quality of life and outcomes of patients with BBS. This case report has been reported in accordance with the CARE criteria (9).

## Case presentation

**Figure 1:**
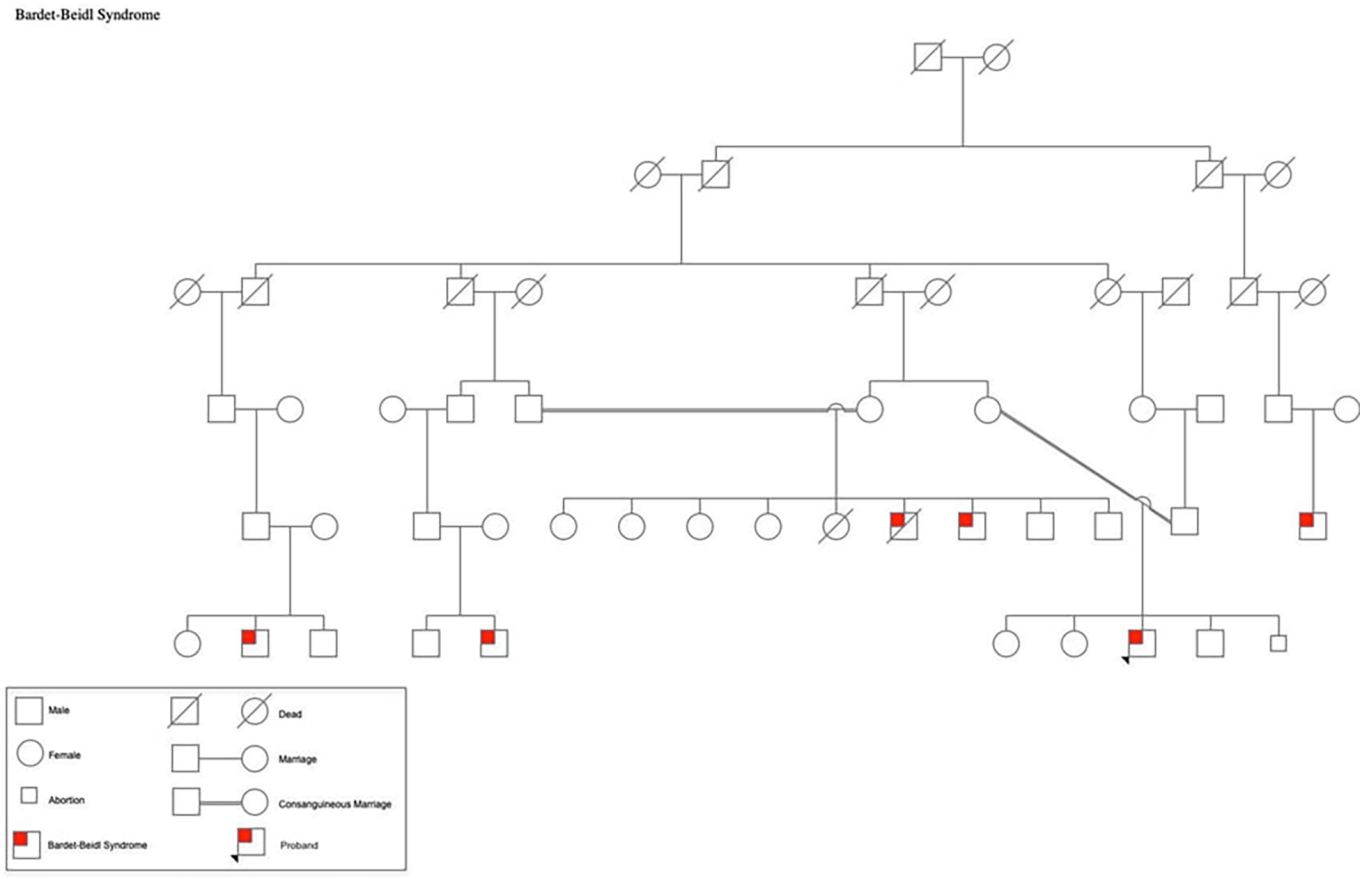
Pedigree of the patient's family showing multiple members affected by BBS.

A 19-year-old Palestinian male born to consanguineous parents with a family history of Bardet-Biedl Syndrome (BBS) (Figure 1) presented to the outpatient clinic for a follow-up appointment for diabetes and obesity. He was born at full-term via normal vaginal delivery, weighing 4.250 kg at birth. He has a history of Hirschsprung's disease that was diagnosed shortly after birth. Additionally, he exhibited postaxial polydactyly in his left hand and feet, as well as syndactyly in the fourth and fifth fingers of his left hand; he also had cryptorchidism. Since childhood he had been suffering from poor vision that worsens during the night so he was diagnosed with retinitis pigmentosa and astigmatism. Moreover, he had complained from rapidly increasing weight and polyuria and was subsequently diagnosed with type 2 diabetes mellitus and hypoparathyroidism. The patient has a family history of five first-degree cousins affected by BBS who experienced nearly the same symptoms. He underwent bilateral orchiopexy for cryptorchidism, and surgical interventions for postaxial polydactyly by removing the extra digit as well as for syndactyly by releasing webbings.

The patient was diagnosed based on BBS Clinical criteria at age 12, as he exhibited 4 primary features; the obesity and polydactyly he had since infancy, red cone dystrophy that was diagnosed by ophthalmologist, in addition to a decrease in the size of the right kidney and mild hydronephrosis in the left kidney that were revealed by ultrasound with normal creatinine level back then (0.9 mg/dl). He also exhibited secondary features including astigmatism, brachydactyly, diabetes mellitus, and a high arched palate.

The patient had so many features that verifies the diagnosis based on the criteria yet there was no hypogonadism or learning disabilities. He did not show signs of cognitive deficits or developmental delay. Additionally, he did not exhibit any speech abnormalities, ataxia, imbalance, spasticity, dental crowding, congenital heart disease or hepatic fibrosis (Table 1).

**Table 1:** Previously reported features of BBS and the positive and negative findings in our patient.

Previously reported BBS features	Present/absent in our case
Primary features	
Retinal dystrophy	Present
Polydactyly	Present
Renal structural abnormalities	Present
Obesity	Present
Hypogonadism in males	Absent
Learning difficulties	Absent
Secondary features	
Diabetes mellitus	Present
High arched palate/missing teeth/dental crowding/short roots	Present
Brachydactyly/syndactyly	Present
Astigmatism/cataract/strabismus	Present
Nephrogenic diabetes insipidus	Absent
Developmental delay	Absent
Speech defect or delay	Absent
Mild spasticity (mainly lower limbs)	Absent
Imbalance/poor coordination/ataxia	Absent
Hepatic fibrosis	Absent
Left ventricular hypertrophy/congenital heart disease	Absent

To confirm the diagnosis and to identify the potential underlying genetic mutations associated with BBS in this case, which is one of the first few cases of the syndrome in Palestine, whole-exome sequencing was performed. The analysis revealed two heterozygous missense variants in the *FBN3* gene (chr19:8190857 G > T, p.R884S, and chr19:8210748 T > A, p.T142S), which has been previously associated with autosomal recessive BBS. However, these variants did not fully segregate with the patient's phenotypic features, indicating the potential involvement of additional genetic factors or modifier genes. A homozygous missense variant in the *BBS2* gene (chr16:56,548,501 C > T, p.S70N), which is one of the Bardet Biedl syndrome (BBS) gene family, was also identified.

As part of his treatment, he uses artificial eye gel and takes 600 mg of calcium carbonate and 850 mg of metformin twice daily. Additionally, he uses topical anti-bacterial and anti-fungal creams, antibiotics and NSAIDs as needed.

**Figure 2:**
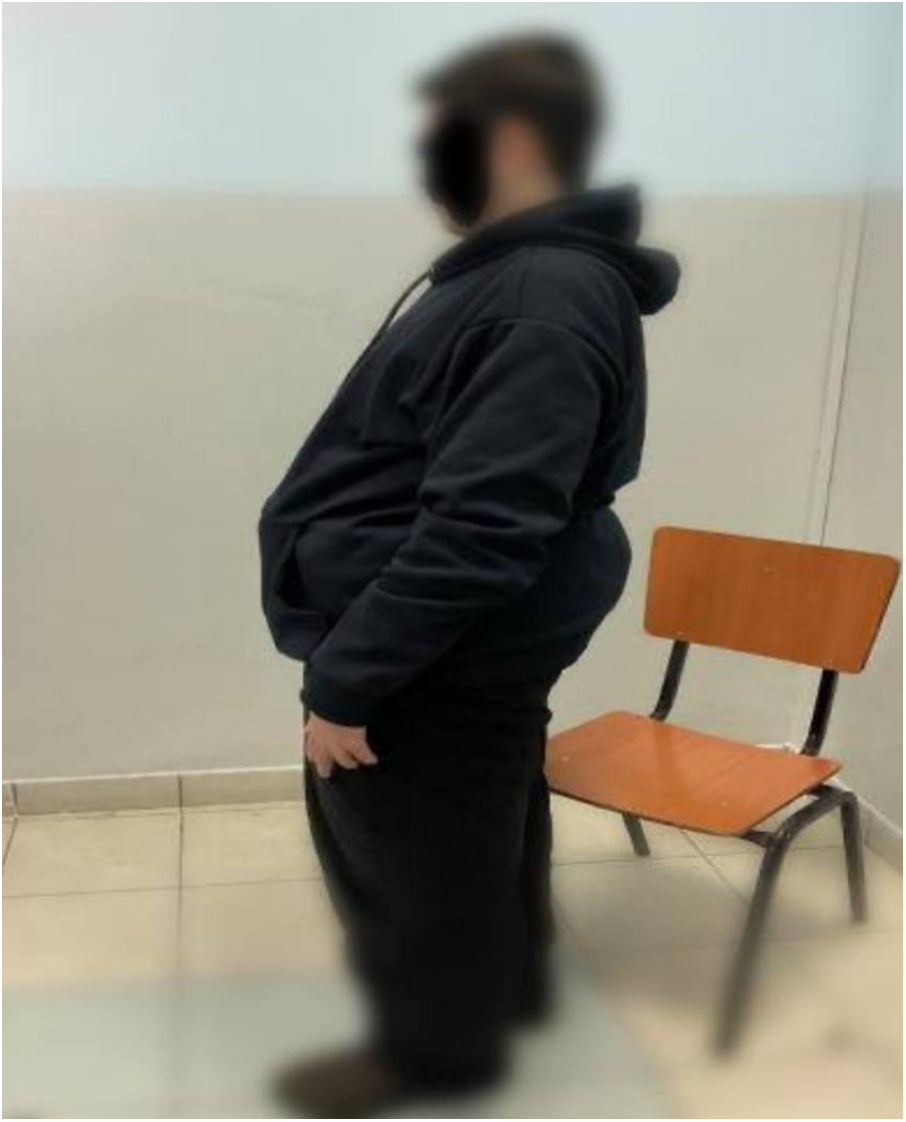
Truncal obesity in a 19-year-old male patient with Bardet-Biedl syndrome.

**Figure 3:**
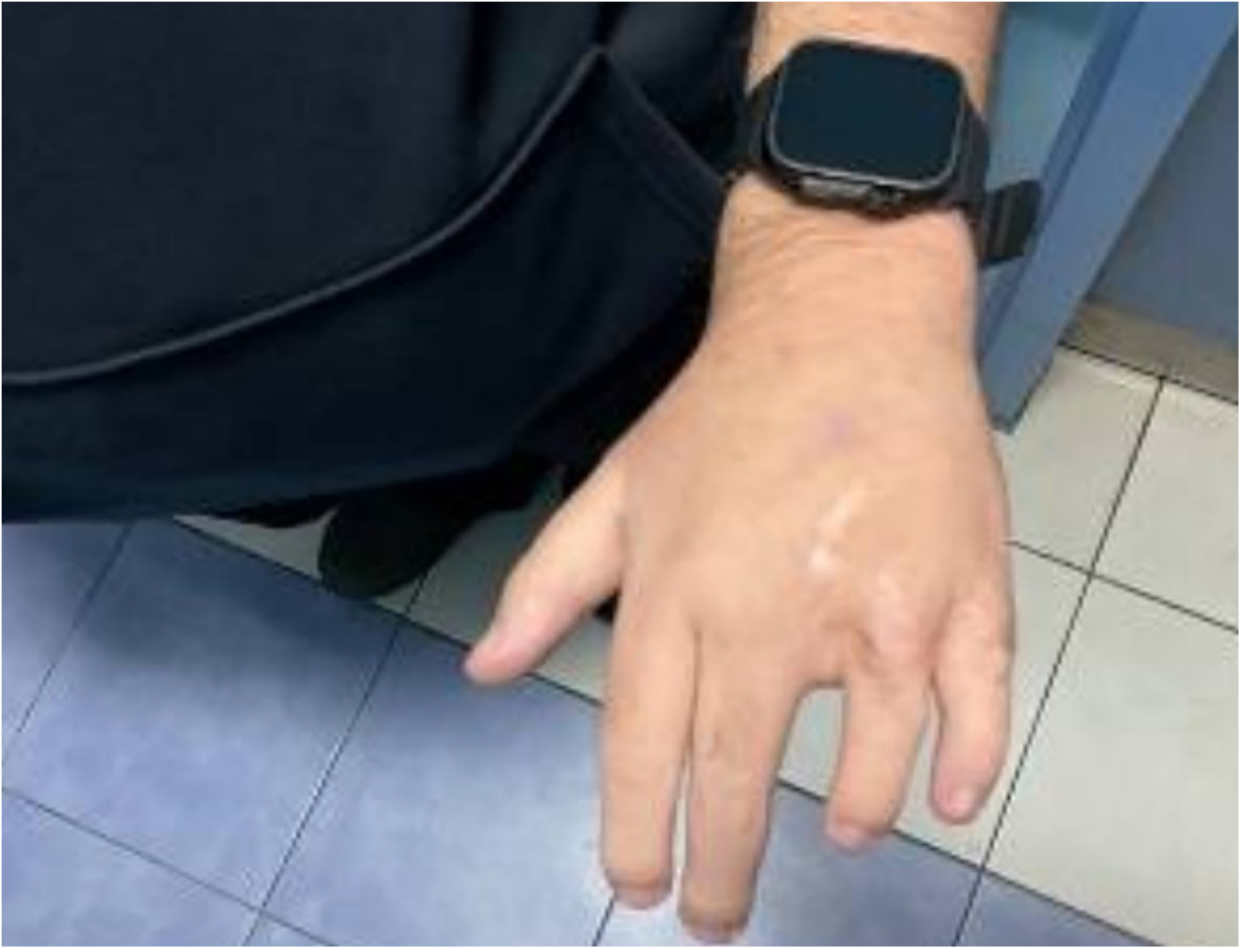
Brachydactyly in the fourth and fifth fingers of the patient's left hand.

**Figure 4:**
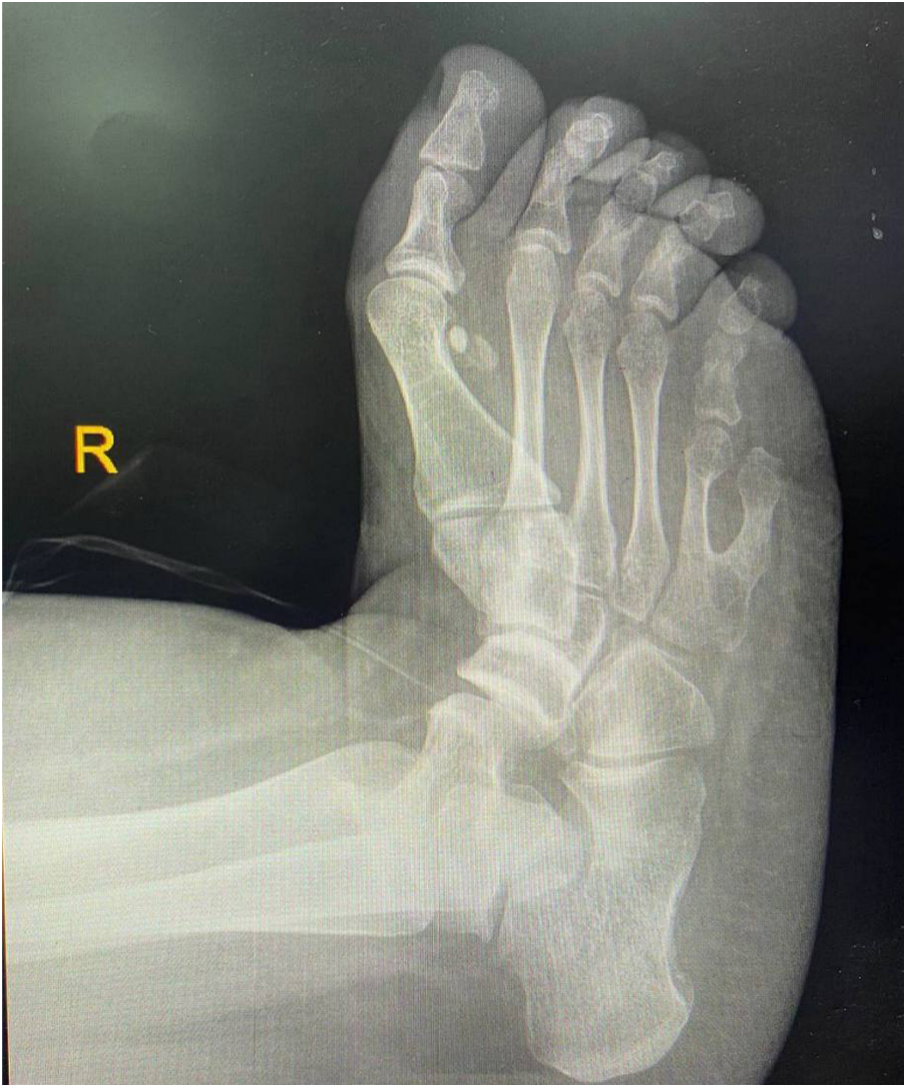
Radiograph demonstrating rocker bottom foot deformity.

Following the diagnosis of BBS, the patient saw a pediatric endocrinologist for follow-ups every three months. On his last visit for follow up on obesity and DM, the patient had poor vision, blurriness, and night blindness. Clinical examination revealed a current weight of 124 kg and a height of 1.64 m, with a BMI of 46 kg/mÂ^2^, indicating evident central obesity (Figure 2), brachydactyly in the fourth and fifth fingers of the left hand (Figure 3), bilateral genu varum, a high-arched palate, acanthosis nigricans, and gynecomastia. A rocker bottom foot was also observed (Figure 4) and blood pressure was 138/72 mmHg. The follow-up investigations conducted by the nephrologist revealed elevated creatinine (1.3 md/dl), accompanied by the presence of 180 mg/24 h of proteinuria, therefore, he considered adding ramipril 1.25 mg once daily to the patient's daily medications. No additional diagnoses were made, and the patient's primary concerns were obesity and diabetes mellitus. The plan involved implementing dietary and lifestyle changes, regularly consulting with an nephrologist, orthopedist, and ophthalmologist, and undergoing bariatric surgery.

## Discussion

BBS is a rare autosomal recessive disorder that exhibits both clinical and genetic diversity (5). The genetic basis of BBS involves mutations in at least 21 known BBS-causing genes, including the BBS gene family (*BBS1*-*BBS20*) and *NPHP1* gene which encodes Nephrocystin-1 protein, each of which contributes to the proper functioning of primary cilia. *BBS1* and *BBS10* are recognized as the primary genes associated with BBS, with mutations in these genes detected in more than 20% of diagnosed cases (3). These mutations disrupt the normal structure and function of cilia, leading to the multisystemic manifestations observed in individuals with BBS. The exact mechanisms by which these genetic mutations result in the specific clinical features of BBS are still being elucidated, but it is clear that primary cilia dysfunction is central to the pathophysiology of the syndrome (10). The prevalence of BBS in North America and Europe ranges from approximately 1 in 140,000 to 1 in 160,000 live births. In contrast, the prevalence rate in the Arab world, specifically in Kuwait, is significantly higher at approximately 1 in 13,000 live births (11). This notable increase in incidence within the Arab world and the broader Middle East region can be attributed to the prevalence of consanguineous marriage, which is commonly observed in this geographical area.

The typical clinical manifestations of BBS encompass various manifestations such as post-axial polydactyly, obesity, retinal dystrophy, learning difficulties, renal abnormalities, genitourinary malformations, cardiovascular involvement, developmental delay, diabetes mellitus, and strabismus. The diagnostic criteria for BBS require either the presence of four major characteristics or a combination of three primary and two secondary features. The primary features include truncal obesity, polydactyly, retinitis pigmentosa, learning disabilities, renal malformations, genital abnormalities (in females), and hypogonadism (in males). The secondary features of BBS encompass a broad spectrum of abnormalities spanning various areas such as oral and dental health, including hypodontia and microdontia. Neurological abnormalities are also observed, such as epilepsy, ataxia, and speech abnormalities. Olfactory dysfunctions, such as anosmia and hyposmia, can also occur. Furthermore, cardiovascular and thoracoabdominal anomalies, including congenital heart disease and Hirschsprung disease, may manifest. Inflammatory bowel and celiac diseases can affect the gastrointestinal system. Additionally, endocrine and metabolic abnormalities such as type 2 diabetes, hypothyroidism, and polycystic ovary syndrome may be observed as well (3, 7, 8, 10–13). Our patient was presented with obesity, polydactyly, retinitis pigmentosa, renal failure, and cryptorchidism as primary features. Regarding secondary features, our patient had type II diabetes mellitus, syndactyly, and bilateral astigmatism and thus he fulfilled the diagnostic criteria for BBS. Notably, a rocker bottom foot was also observed in this patient, which is an atypical finding for BBS as it is not commonly reported in the literature. Our patient does not have hypogonadism or learning disabilities, and despite being common in 62% of BBS cases, he did not exhibit any developmental delay or cognitive deficits. Furthermore, he showed no signs of speech abnormalities, which 60% of patients experience. He does not suffer from congenital heart disease, hepatic fibrosis, ataxia, imbalance, spasticity, or dental crowding.

Most symptoms associated with BBS become apparent after several years of development and are not observable in early childhood. Hence, diagnosing BBS in young children poses difficulties owing to the progressive nature of its clinical characteristics (10). In a noteworthy population-centric study conducted in the United Kingdom, the mean age at BBS diagnosis was determined to be nine years (14). Following diagnosis, it is important to conduct a comprehensive assessment to determine disease severity, including urine analysis, renal function tests, blood sugar levels, abdominal ultrasound, ophthalmic evaluation of visual acuity and field defects, examination of genitalia, obesity assessment through BMI calculation, cardiac evaluation, neurological examination, and hearing evaluation (15). Genetic counseling can offer advantages to both affected individuals and their families, and regular follow-up is essential. Our patient was diagnosed at the age of 12 years, which can be considered a delayed diagnosis compared to other cases in the literature, especially considering the fact that a number of his cousins were also diagnosed with the syndrome.

Numerous renal abnormalities have been documented in the literature concerning BBS, including unilateral agenesis, chronic kidney disease (CKD), parenchymal cysts, dysplastic kidneys, renal scarring, calyceal clubbing, fetal lobulation, renal calculi, and vesicoureteral reflux (10). The progression and prognosis of renal disease in patients with BBS remains unclear. Renal dysfunction can arise from primary causes, such as cystic renal disease, as well as secondary causes, such as hypertension, diabetes, and metabolic syndrome. Renal failure is the leading cause of mortality among individuals with BBS, accounting for approximately 25% of deaths by the age of 44 years (4).

Given the multisystem nature of BBS and that the management is primarily supportive and aimed at halting the development of complications while improving the quality of life of patients, a multidisciplinary approach involving comprehensive evaluation and ongoing monitoring of various organ systems, with a focus on addressing the specific clinical manifestations and complications associated with the syndrome in each diagnosed case, is strongly required. Genetic counseling is also essential for patients and their families to understand the genetic basis of the condition, assess recurrence risks, and make informed decisions regarding family planning.

Therapeutic choices for BBS are based on the findings and may include conservative or surgical interventions. Obesity management involves lifestyle modification, dietary education, and exercise, in addition to addressing metabolic syndrome and other obesity-related issues. In resource-limited areas, however, managing nutrition for individuals with BBS is challenging due to shortage of well-trained dietitians and the presence of blindness, which significantly impacts nutrition management. Speech therapy may be beneficial for individuals with delayed speech and learning disabilities. Surgical correction may be necessary to prevent gonadal complications. For females, contraceptive advice should be offered rather than assuming infertility is likely, and hormone replacement therapy may be required for women with hormone imbalances. Patients with renal failure can be treated with hemodialysis or peritoneal dialysis, and renal transplantation may be an option for chronic renal failure (15). Surgical removal of accessory digits can be considered for aesthetic purposes. Regular consultation with an ophthalmologist is recommended to correct refractive errors. The use of low-vision aids can be beneficial for visually impaired patients (14). Early educational strategies, such as teaching Braille, mobility training, adapting living skills, and improving computer abilities, should be implemented for individuals at risk of blindness as there is no definitive treatment for the visual loss associated with BBS (16).

The significance of genotype-phenotype correlations is evident in this case, as mutations in each of *FBN3* and *BBS2* genes resulted in distinct clinical presentations. The identification of mutations in these genes suggests a potential link between ciliary dysfunction, connective tissue abnormalities, and the pathogenesis of BBS. It also supports the notion that BBS is a genetically heterogeneous disorder with overlapping phenotypes and highlights the need for comprehensive genetic screening in patients with atypical presentations of BBS, as mutations in genes beyond the traditionally associated BBSome complex may contribute to the disease. Understanding the functional consequences of these mutations can guide personalized management strategies and pave the way for targeted therapies.

BBS is traditionally associated with ciliary dysfunction due to mutations in genes that form the BBSome complex, of which *BBS2* is a critical component. The identification of *BBS2* mutations reaffirms the importance of ciliary pathways in the pathogenesis of BBS, leading to characteristic features such as retinal dystrophy, obesity, polydactyly, and renal anomalies (17). The involvement of *FBN3*, a gene encoding fibrillin-3, introduces a novel aspect to the understanding of BBS as fibrillins are known to play a crucial role in the formation of extracellular microfibrils that provide structural support and elasticity to connective tissues (18). Mutations in *FBN3* have been associated with skeletal dysplasias and connective tissue abnormalities, which aligns with the skeletal and renal manifestations observed in this patient.

Overall, this case report contributes to our evolving understanding of BBS and underscores the importance of genetic analysis in diagnosing and managing the syndrome. It highlights the need for continued research to elucidate the molecular mechanisms underlying BBS and explore potential therapeutic interventions. By unraveling the complexities of BBS genetics and its associated pathways, we can strive towards improved outcomes and quality of life for affected individuals. However, this case report has certain limitations. Firstly, case reports, by their nature, do not allow for the establishment of causality or generalization to the broader population due to the lack of control groups and the unique specificity of individual cases. Additionally, the rarity of BBS and the even less common occurrence of *FBN3* mutations within this context mean that our findings may not be representative of all BBS presentations. Another limitation is the potential for bias, as case reports often focus on novel or unusual presentations that may not reflect the typical disease course. Furthermore, the retrospective design of case reports can lead to selective reporting and recall bias. Lastly, while this report provides a detailed account of one patient's genetic profile and clinical progression, it does not offer a comprehensive view of the variability and full spectrum of BBS, which would require a larger cohort study or systematic review for more robust conclusions. These limitations highlight the need for cautious interpretation of the findings and suggest areas for future research to build upon the knowledge presented here.

## Conclusion

BBS is a rare, heterogeneous ciliopathy that affects multiple organs and systems. It is mainly diagnosed based on clinical criteria and can be confirmed using genetic testing. Early diagnosis and multidisciplinary management of BBS are essential to mitigate or delay the occurrence of complications and to enhance the overall well-being of patients. Further research is warranted to investigate the potential genetic factors contributing to the complex manifestations of BBS and to enhance our understanding of this rare genetic disorder.

## Data Availability

The original contributions presented in the study are included in the article. Further inquiries can be directed to the corresponding author/s.

## References

[1] Bardet G. On congenital obesity syndrome with polydactyly and retinitis pigmentosa (a contribution to the study of clinical forms of hypophyseal obesity) thesis, for the degree of doctor of medicine. Obes Res. (1995) 3(4):387–99. 10.1002/j.1550-8528.1995.tb00165.x 8521156

[2] Biedl A. A pair of siblings with adiposo-genital dystrophy. Obes Res. (1995) 3(4):404–404. 10.1002/j.1550-8528.1995.tb00167.x 8521158

[3] Mandal RK Pande R Kc RS Acharya B. Bardet-Biedl syndrome: a case report from Nepal. Asian J Med Sci. (2021) 12(8):158–63. 10.3126/ajms.v12i8.38262

[4] Forsythe E Beales PL. Bardet-Biedl syndrome. Eur J Hum Genet. (2013) 21(1):8–13. 10.1038/ejhg.2012.115 22713813 PMC3522196

[5] Katsanis N Ansley SJ Badano JL Eichers ER Lewis RA Hoskins BE Triallelic inheritance in Bardet-Biedl syndrome, a mendelian recessive disorder. Science. (2001) 293(5538):2256–9. 10.1126/science.1063525 11567139

[6] Hassan S Khan QA Saravanan P Iram S Rohail S Belay NF Megaloblastic anemia in Bardet-Biedl syndrome: a rare case report. Clin Med Insights Case Rep. (2023) 16:11795476231193896. 10.1177/11795476231193896 37588947 PMC10426295

[7] Toledo NB Maimone JBR Marcos AAA Leite EHM de S A Junior C. Bardet-Biedl syndrome: case series and literature revision. Rev Bras Oftalmol. (2018) 77(6):360–2. 10.5935/0034-7280.20180079

[8] Madireddi J Acharya V Suryanarayana J Hande HM Shetty R. Bardet-Biedl syndrome: multiple fingers with multiple defects!. BMJ Case Rep. (2015) 2015:bcr2015211776. 10.1136/bcr-2015-211776 26611481 PMC4680628

[9] Agha RA Franchi T Sohrabi C Mathew G Kerwan. The CARE 2020 guideline: updating consensus surgical case report (CARE) guidelines. Int J Surg. (2020) 84:226–30. 10.1016/j.ijsu.2020.10.034 33181358

[10] Elawad OAMA Dafallah MA Ahmed MMM Albashir AAD Abdalla SMA Yousif HHM Bardet-Biedl syndrome: a case series. J Med Case Rep. (2022) 16(1):169. 10.1186/s13256-022-03396-6 35484558 PMC9052695

[11] M’hamdi O Ouertani I Maazoul F Chaabouni-Bouhamed H. Prevalence of Bardet-Biedl syndrome in Tunisia. J Community Genet. (2011) 2(2):97–9. 10.1007/s12687-011-0040-6 22109794 PMC3186025

[12] Valverde D Castro-Sánchez S Álvarez-Satta M. Bardet-Biedl syndrome: a rare genetic disease. J PediatrGenet. (2015) 2(2):077–83. 10.3233/PGE-13051 PMC5020962 27625843

[13] Osman F Iqbal MI Islam MN Kabir SJ. Bangladeshi case series of Bardet-Biedl syndrome. Case Rep Ophthalmol Med. (2023) 2023:1–7. 10.1155/2023/4017010 PMC10122572 37096247

[14] Beales PL Elcioglu N Woolf AS Parker D Flinter FA. Original articles new criteria for improved diagnosis of Bardet-Biedl syndrome: results of a population survey. J Med Genet. (1999) 36(6):437–46. 10874630 PMC1734378

[15] Sowjanya B Sreenivasulu U Naidu JN Sivaranjani N. End stage renal disease, differential diagnosis, a rare genetic disorder: Bardet-Biedl syndrome: case report and review. Indian J Clin Biochem. (2011) 26(2):214–6. 10.1007/s12291-011-0116-4 22468053 PMC3107414

[16] Dervisoglu E Isgoren S Kasgari D Demir H Yilmaz A. Obesity control and low protein diet preserve or even improve renal functions in Bardet-Biedl syndrome: a report of two cases. Med Sci Monit. (2011) 17(1):CS12–4. 10.12659/MSM.881320 21169913 PMC3524693

[17] Kleinendorst L Alsters SIM Abawi O Waisfisz Q Boon EMJ van den Akker ELT Second case of Bardet-Biedl syndrome caused by biallelic variants in IFT74. Eur J Hum Genet. (2020) 28(7):943–6. 10.1038/s41431-020-0594-z 32144365 PMC7316806

[18] Genovesi ML Torres B Goldoni M Salvo E Cesario C Majolo M Case report: a novel homozygous missense variant of *FBN3* supporting it is a new candidate gene causative of a Bardet-Biedl syndrome–like phenotype. Front Genet. (2022) 13:924362. 10.3389/fgene.2022.924362 35910214 PMC9334770

